# Highly Air Stable Tin Halide Perovskite Photovoltaics using a Bismuth Capped Copper Top Electrode

**DOI:** 10.1002/advs.202301497

**Published:** 2023-06-16

**Authors:** Anjana Wijesekara, Yisong Han, David Walker, Steven Huband, Ross Hatton

**Affiliations:** ^1^ Department of Chemistry University of Warwick Coventry CV4 7AL United Kingdom; ^2^ Department of Physics University of Warwick Coventry CV4 7AL United Kingdom

**Keywords:** bismuth, cathodes, stability, tin perovskite photovoltaic, tin perovskite solar cells

## Abstract

An effective approach is reported to enhance the stability of inverted organo‐tin halide perovskite photovoltaics based on capping the cathode with a thin layer of bismuth. Using this simple approach, unencapsulated devices retain up to 70% of their peak power conversion efficiency after up to 100 h testing under continuous one sun solar illumination in ambient air and under electrical load, which is exceptional stability for an unencapsulated organo‐tin halide perovskite photovoltaic device tested in ambient air. The bismuth capping layer is shown to have two functions: First, it blocks corrosion of the metal cathode by iodine gas formed when those parts of the perovskite layer not protected by the cathode degrade. Second, it sequesters iodine gas by seeding its deposition on top of the bismuth capping layer, thereby keeping it away from the electro‐active parts of the device. The high affinity of iodine for bismuth is shown to correlate with the high polarizability of bismuth and the prevalence of the (012) crystal face at its surface. Bismuth is ideal for this purpose, because it is environmentally benign, non‐toxic, stable, cheap, and can be deposited by simple thermal evaporation at low temperature immediately after deposition of the cathode.

## Graphical abstract

Unencapsulated inverted organo‐tin halide perovskite photovoltaics using a bismuth capped copper cathode achieve record stability when tested in ambient air under 1 sun simulated solar illumination and electrical load. The bismuth layer blocks corrosion of the copper electrode by iodine gas and sequesters iodine gas by seeding its condensation on top of the device.

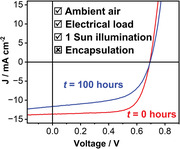

## 1 Introduction

In recent years there has been rapidly growing interest in research into tin halide perovskites as the light harvesting semiconductor in photovoltaic (PV) devices.^[^ ^1^, ^2^ ^]^ The Achilles’ heel of tin perovskites for PV applications is their susceptibility to oxidation in air, which stems from the tendency of Sn^2+^ to convert to the more thermodynamically stable Sn^4+^ oxidation state upon exposure to ambient air.^[^ ^3^ ^]^ Whilst for practical application PV devices are encapsulated to slow moisture and oxygen ingress, over the long operational life of a PV device moisture and oxygen ingress are inevitable, particularly for those supported on flexible substrates. Lanzetta et al. have shown that in organo‐tin halide perovskites the degradation products are I_2_, SnI_4_, and AI where A depends on the choice of organic A‐site cation and is usually formamidinium (CH_5_N_2_^+^) or guanidinuim (CH_6_N_3_^+^).^[^ ^4^, ^5^ ^]^ SnI_4_ subsequently reacts with solid AI to form double perovskite A_2_SnI_6_ or hydrolyses to form SnO_2_ and HI, the latter of which reverts to I_2_ gas upon reaction with moisture in the air, which has been shown to be a major source of degradation in organo‐tin halide perovskite PVs (PPVs).^[^ ^4^, ^5^, ^6^ ^]^

We have recently reported record stability for an unencapsulated organo‐tin halide PPV device in ambient air, under constant one sun simulated solar illumination and under electrical load when using copper (Cu) as the cathode in place of a conventional silver (Ag) electrode, together with a bathocuproine (BCP) electron extraction layer: Under this test condition the power conversion efficiency (*η*) of the champion device reduced to 70% of its peak value after 44.5 h testing.^[^ ^6^ ^]^ In that work, it was shown that a major source of device degradation is the corrosive effect of iodine containing volatile small molecules, particularly iodine (I_2_) gas, which corrode the metal cathode from the top surface rather than the buried interface.^[^ ^6^ ^]^ This mechanism of degradation is particularly pronounced for organo‐tin halide PPVs because of the much faster degradation of organo‐tin perovskites in the air than their lead analogues.^[^ ^4^, ^5^, ^6^ ^]^ Consequently, those parts of the perovskite film not protected by the cathode degrade quickly upon exposure to ambient air producing a cloud of I_2_ gas that aggressively corrodes the metal cathode from its top surface.^[^ ^6^ ^]^ I_2_ has also been shown to catalyze the degradation of organo‐tin halide perovskite itself and so it is important to ensure that I_2_ gas formed in the aforementioned way does not find its way into the photo‐active parts of the device.^[^ ^4^, ^5^ ^]^ I_2_ gas is particularly problematic, because it is volatile at the typical operating temperatures of PPVs: 20–60 °C.

A logical strategy to protect the top metal electrode from I_2_ gas is to cap it with a layer that does not react with I_2_ below ≈60 °C. However, the propensity of almost all metals to react with halogen species produced when halide perovskites degrade is well established and is a critical concern for the long‐term stability of PPVs, even with device encapsulation.^[^ ^7^ ^]^ I_2_ is also known for its facile diffusion into molecular and polymer films, and through pinholes and along grain boundaries in oxide films, so the capping layer must also be capable of blocking diffusion of I_2_.^[^ ^7^, ^8^, ^9^, ^10^, ^11^ ^]^ When these two basic requirements are combined with the commercial need for low raw material cost and amenability to processing using low‐cost large area deposition methods, the choice of capping material becomes surprisingly limited. I_2_ gas is a powerful oxidizing agent that aggressively corrodes most metals at low temperature due to a negative standard enthalpy of formation of the metal iodide and no energetic barrier to breaking the metal‐metal bond to form the metal‐iodine bond: See Table S1 (Supporting Information) for a list of 17 technologically important metals. Notable exceptions to this are the molybdenum (Mo), bismuth (Bi) and gold (Au). While Mo is widely used for its anti‐corrosive properties, the energy outlay for deposition of Mo thin films is very high, which requires that its deposition by sputtering or electron‐beam evaporation. For example, to achieve a vapor pressure of 10^−4^ Torr Mo must be heated to 2117 °C – more than three times higher than that of Ag (832 °C).^[^ ^12^ ^]^ Au is widely used as an electrode in research laboratories, but is prohibitively expensive for practical application in PPVs.^[^ ^13^ ^]^ Conversely, Bi is ≈0.01% of the cost of Au^[^ ^13^ ^]^ and so is sufficiently low cost for application in PPVs. Unlike Mo, Bi can be deposited using conventional thermal evaporation because a vapor pressure of 10^−4^ Torr can be achieved for a source temperature of 517 °C. Bi does not react with I_2_ gas below 150 °C,^[^ ^14^ ^]^ which is well above the temperature a PPV would be expected to operate.

Bismuth (Bi), which is considered to be the heaviest stable atom (atomic number 83),^[^ ^15^ ^]^ has five valence electrons (*s^2^p^3^*) but the large separation between the *s* and *p*‐orbital energy levels means that it generally behaves like an element with three valence electrons (i.e.*, p^3^*). To the author's knowledge, to date there has been only one report of using elemental Bi in the context of a PPV device.^[^ ^15^ ^]^ In 2019 Wu *et al*.^[^ ^16^ ^]^ showed that the stability of inverted lead halide PPVs using a conventional Ag cathode could be improved by inserting a thin layer of Bi between the Ag cathode and BCP electron extraction layer. Bi was chosen because there is a significant barrier to reaction between I_2_ and Bi (18.3 kJ mol^−1^) and this semi‐metal is sufficiently electrically conductive for inclusion of a 20 nm thick layer at this interface without significantly contributing to device series resistance. They showed that the Bi layer blocked the diffusion of iodide ions from the underlying perovskite to the Ag electrode and also blocked the diffusion of Ag into the perovskite layer.^[^ ^16^ ^]^ In that study, the stability of devices tested in air was not reported and the buried Bi layer could not have protected the top surface of the Ag electrode from corrosion.

Herein we report a two‐fold improvement on the previous record stability for unencapsulated organo tin halide PPV devices tested under 1 sun solar illumination in ambient air and under electrical load. The key to achieving this large improvement is capping the Cu cathode with an evaporated Bi layer deposited immediately after Cu deposition. We show that the Bi layer not only serves to block corrosion of the Cu cathode by I_2_ gas emanating from those parts of the perovskite layer not buried beneath the cathode, but also sequesters I_2_ gas by nucleating its deposition on top of the Bi layer and shed light on the underlying reason for this finding. Bi is an ideal choice for iodine blocking and capture in PPVs because it is environmentally benign, nontoxic, stable, and relatively cheap at ≈3% of the cost of Ag.^[^ ^17^ ^]^ While testing unencapsulated PPV devices in ambient air is clearly an accelerated degradation test, because for practical application PV devices would always be encapsulated and so the rate of air ingress is very slow, accelerated degradation tests are useful because they help researchers to quickly identify promising materials and device designs needed to achieve long device life. In recent years several chemical approaches to improving the stability of organo‐tin halide perovskites toward degradation in air have been reported,^[^ ^18^, ^19^, ^20^, ^21^, ^22^, ^23^ ^]^ which have translated to impressive stability of up to 1000 h when stored in the dark in air,^[^ ^18^, ^19^, ^20^, ^21^ ^]^ and hundreds of hours when white‐light soaked in air.^[^ ^18^, ^19^, ^21^ ^]^ However, these test conditions do not capture the full set of degradation processes that can occur in an operational PPV device (i.e., a device doing electrical work).^[^ ^24^ ^]^ Surprisingly few reports relate to the test conditions of 1 sun solar illumination under electrical load,^[^ ^6^, ^22^ ^]^ which is the test condition of most practical relevance for a photovoltaic device^[^ ^24^ ^]^ and the test condition used in the current study.

## 2 Results and Discussion

For the purpose of the device study, model tin halide PPV devices with the structure; ITO|PEDOT:PSS (Al 4083)| FA_0.78_GA_0.2_SnI_3_ ‐1 mol % EDAI_2_ + 10 mol % SnF_2_|C_60_ (32.5 nm)|BCP (5 nm)|Cu (100 nm)|Bi) were fabricated using an adaptation of the method reported by Jokar et al.,^[^ ^20^ ^]^ where PEDOT:PSS is the conventional hole‐extraction layer poly(3,4‐ethylenedioxythiophene) polystyrene sulfonate. In order to determine the minimum Bi thickness needed to passivate the 100 nm Cu cathode toward corrosion by I_2_ gas in ambient air, an adaptation of the optical calcium – oxygen corrosion test^[^ ^25^ ^]^ was performed: Cu films with a thickness of 100 nm capped with a Bi layer of varying thickness were deposited onto glass substrates. The edges of the bilayer films were then sealed with epoxy resin to prevent Cu film corrosion from underneath the Bi layer resulting from I_2_ ingress through the exposed edge of the Cu film where it meets the edge of the supporting glass substrate. The sublimating property of iodine crystals at ambient pressures enables the facile formation of I_2_ gas in air close to room temperature. The iodine crystals were heated to ≈45 °C to produce a continuous flow of I_2_ gas. Cu films with a Bi overlayer thickness of 1 nm, 5 nm, 25 nm, 35 nm, and 50 nm were simultaneously exposed to the same I_2_ vapor. From the time taken for the opaque reflective red / brown color characteristic of Cu metal to be converted to CuI – which is optically transparent^[^ ^26^ ^]^ – a Bi thickness of ≥ 35 nm was found to impart optimal protection of the underlying Cu (Figure S1, Supporting Information). Notably, if the edges of the glass slide were not sealed with epoxy resin conversion of the Cu metal into CuI begins within minutes regardless of the Bi overlayer thickness, starting from the edges and progressing toward the center, which evidences the ability of I_2_ gas to aggressively diffuse through narrow gaps – in this case through the 100 nm thickness of the Cu film.

All devices were tested under continuous 1 sun simulated illumination using a class AAA Xenon‐arc lamp solar simulator with devices operating at, or very close to, the maximum‐power‐point and were not encapsulated. Each device has an active area of 6 mm^2^. The temperature of the devices stabilized at ≈43 °C and the relative humidity fluctuated in the range 30%–70%. We have previously reported that for organo‐tin halide PPVs using a Cu cathode the efficiency improves dramatically during the first hours of testing due to a sharp increase in open‐circuit voltage (*V_OC_*) and device fill‐factor (*FF*),^[^ ^6^ ^]^ and the reasons for these transient effects have been discussed previously.^[^ ^6^, ^27^ ^]^ Other research groups have also reported that organo‐tin PPVs need to be aged by storing or light soaking for several days under nitrogen to achieve optimal *η*.^[^ ^20^, ^22^ ^]^ The focus of the current manuscript is the operational stability of the devices after the point at which these transient improvements in performance parameters have stopped. The starting point for all device lifetimes reported in this paper is therefore the time at which the device achieves its highest *η*.

**Figure 1:**
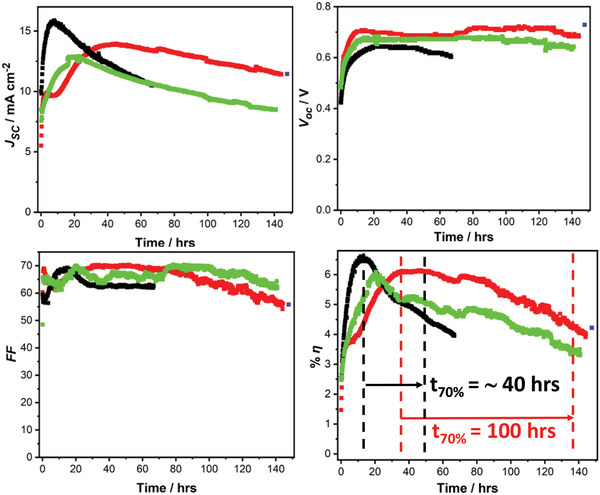
Evolution of the *J_SC_*, *V_OC_*, *FF*, and *η* for the champion unencapsulated PPV devices with the structure ITO|PEDOT:PSS (Al 4083)|perovskite|C_60_|BCP|Cu with (red) and without (black) a 35 nm Bi layer, tested in ambient air under continuous 1 sun simulated solar illumination with the device under load. The last point in the data set for the device with a Bi layer, in blue, corresponds to the performance obtained when the device was re‐tested in a nitrogen atmosphere (i.e., a glove box) immediately after stability testing in air. The arrow in the *η* versus time plot (bottom right) shows that device stability is measured after the point at which the power conversion efficiency peaks. Also shown is the evolution of the *J_SC_*, *V_OC_*, *FF*, and *η* of a Bi capped device with average stability (green).

**Figure** **1** shows the champion device data for both the control (i.e., devices without a Bi layer) and for devices with Bi layer. The stability of the champion control device is in close agreement with that reported previously for devices with the same structure, fabricated in the same way in the same laboratory, but using a different batch of chemicals.^[^ ^6^ ^]^ For the champion device with a Bi layer, *η* peaks after 35 hours and then degrades to 70% of the peak value (t_70_) only after a further 100 h testing. Remarkably, 100% of the peak *η* is retained for 16 h before the onset of degradation. The average t_70_ (± 1 standard deviation) for devices with and without a bismuth capping layer is 85.4 (± 14.6) h and 29.6 (± 10.3) h, respectively. Also included in Figure 1 is a set of data for a Bi capped device with t_70_ stability close to the average and whose peak power conversion efficiency occurs after a similar time period to that of the champion reference device. To our knowledge this stability is more than twice the highest device stability reported to date for an unencapsulated tin halide perovskite PV tested in ambient air under 1 sun constant illumination under electrical load^[^ ^1^, ^2^, ^6^, ^18^, ^19^, ^21^, ^22^ ^]^ (Table S2, Supporting Information). After 100 h of continuous illumination under electrical load there is no significant deterioration in *V_OC_*. Over this period the short‐circuit current (*J_SC_*) and *FF* are reduced by ≈16% and ≈15% respectively. The data in Figures S2 and S3 (Supporting Information) show the evolution of the performance parameters for a larger data set over 145‐h testing (Figure S2, Supporting Information) and also for the shorter period of 80‐h (Figure S3, Supporting Information), and show the full spread in performance parameters. While there is a weak correlation between the time taken to achieve peak power conversion efficiency and t_70_; Figure S4 (Supporting Information), it is too weak to explain the large difference in average and champion t_70_ for devices with and without a Bi capping layer. There is also no correlation between device stability and peak *η*, since devices with a peak *η* of 4.6%, 5.6%, and 6.6% all retain 70% of their peak *η* after ≈100 h testing: Figure S2 (Supporting Information). While the variation of peak *η* is greater than typically reported for organo‐lead halide PPVs, it is not unusual for organo‐tin halide PPVs.^[^ ^6^, ^18^, ^19^, ^21^ ^]^

At the end of the device stability test those areas of perovskite not covered by the cathode have changed from a deep red to a transparent yellow; Figure S5 (Supporting Information), consistent with degradation of the perovskite layer. To determine the extent of degradation of the perovskite film beneath the Bi capped Cu cathode after an extended period of device stability testing in air, the electrode from a device tested for 145 h was delaminated and the BCP and C_60_ layers were washed away using chlorobenzene to reveal the perovskite layer. Elemental analysis was then performed using energy dispersive X‐ray spectroscopy (EDX). The same procedure was performed on a device from the same batch that had not been stability tested (i.e., a fresh device). The Sn:I ratio for the perovskite in the aged and fresh devices is the same within error: 1:1.65 ± 0.14 and 1:1.67 ± 0.09, respectively. (Notably, the Sn:I ratio is much lower than would be expected based on the film composition (i.e., 1:2.8) because the film is supported on an indium‐tin oxide (ITO) coated glass substrate and so the Sn signal from the underlying ITO contributes to the Sn signal intensity.) This close agreement is compelling evidence that the perovskite in the aged device is not deficient of iodine, as would be expected for a perovskite film exposed to ambient air due to loss of iodine in the form of I_2_ gas. This finding is consistent with the observation that the corresponding scanning electron microscopy (SEM) images of the perovskite films with and without device aging show no morphological evidence for degradation of the perovskite: Figure S6 (Supporting Information).

**Figure 2:**
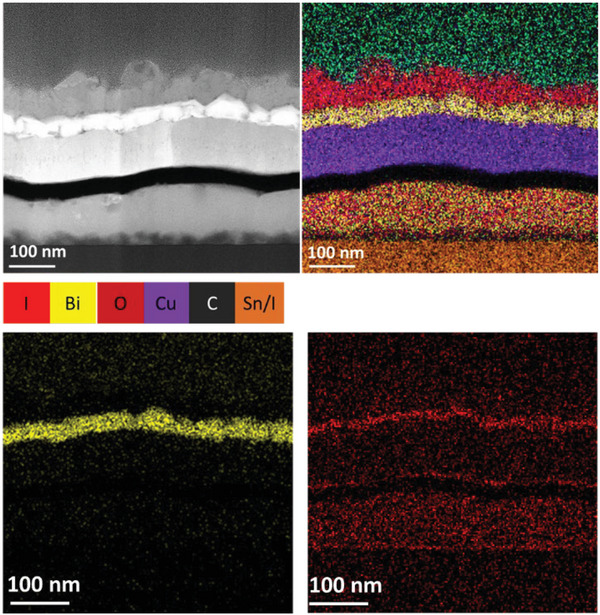
Cross‐sectional TEM image of an aged PPV device with the structure of ITO|PEDOT:PSS (Al 4083)|perovskite|C_60_|BCP|Cu|Bi, and the corresponding EDX elemental analysis.

We have previously shown that degradation of inverted organo‐tin halide PPVs using a Cu cathode tested in ambient air results from reaction of I_2_ gas evolved from the degraded perovskite layer not underneath the cathode with the top surface of the Cu electrode.^[^ ^6^ ^]^ The reaction of Cu with I_2_ to form CuI is most pronounced at the edges of the electrode and the reduction in device *J_SC_* correlated with the reduction in the area of the Cu electrode.^[^ ^6^ ^]^ The exceptional stability of devices using a Cu electrode capped with Bi is therefore attributed to the ability of the Bi layer to resist reaction with I_2_ gas and its ability to block diffusion of I_2_. To validate this hypothesis cross‐sectional transmission electron microscopy (TEM) with spatially resolved EDX analysis of a degraded PPV device was performed. For this experiment devices were fabricated on a silicon wafer instead of ITO coated glass and stored in ambient air in a closed petri‐dish for one week. The petri‐dish was sealed to ensure that the I_2_ gas remained near to the device, as would be the case for a fully encapsulated device. A cross‐section taken from near to the edge of the device (i.e., in closest proximity to source of I_2_ gas) imaged using high resolution TEM and nanoscale EDX is shown in **Figure** **2** and Figure S7 (Supporting Information). A representative EDX line profile is shown in Figure S8 (Supporting Information). These data show that the Bi forms a layer of uniform thickness on top of the Cu cathode and the top few nanometers of the Bi have oxidized. Unexpectedly, there is also a layer of iodine on the top surface of the Bi layer of thickness 40–80 nm.

The presence of a thick layer of iodine on top of aged PPV devices is confirmed by the results of SEM – EDX analysis, since the Sn:I ratio increases from 1:1.96 ± 0.15 to 1:8.3 ± 4.2, with the large error in the latter indicating a large variation in iodine layer thickness across the electrode. Spatially resolved SEM/EDX measurements; Table S3 (Supporting Information) and Figure S9 (Supporting Information), give the Sn:I ratio at the edge and center of the cathode as 1:10.1 (± 3.7) and 1:4.1 (± 0.6), respectively, which shows that the adsorbed iodine layer is thickest near to the edge of the electrode (i.e., nearest the source of I_2_ gas). Notably, the TEM cross‐section shown in Figure 2 was taken from near to the edge of the cathode where, based on the spatially resolved SEM/EDX analysis, the iodine layer is expected to be greatest. These findings show that the Bi capping layer efficiently nucleates the deposition of I_2_ from the gas phase.

The ratio of Bi to O in the very thin oxide layer, determined from nanoscale TEM/EDX analysis; Figure S10 (Supporting Information), is 1: 1.8 (± 0.6). Within the error of the measurement, this ratio is consistent with Bi_2_O_3_ – the most common oxide of metallic Bi. The oxidation of Bi in ambient air is known to be spontaneous,^[^ ^28^ ^]^ but the rate of growth of the oxide layer is limited by the rate of diffusion of Bi ions across the oxide layer in accordance with Wagner's theory^[^ ^29^ ^]^ and so slows dramatically after the first few nanometers of oxide formation.^[^ ^30^ ^]^ While the nano‐scale EDX analysis indicates that there is ≈2.4 at% iodine in the oxide layer, this is deemed to be within the error of the measurement because the oxide layer is extremely thin and immediately adjacent to a much thicker layer of iodine. It is therefore likely that the small iodine signal results from the adjacent iodine layer due to broadening of the electron beam. Crucially, Bi_2_O_3_ does not react with I_2_ below 200 °C^[^ ^31^ ^]^ and so its formation is not detrimental to the ability of the Bi layer to protect the underlying Cu from corrosion by I_2_. The absence of Sn in the I_2_ layer (Figures S7 and S10, Supporting Information) is evidence that SnI_4_ produced during perovskite decomposition in air plays a minor role in corroding the Cu electrode, which is consistent with its lower volatility as compared to I_2_ and its facile decomposition of SnI_4_ into I_2_ in ambient air via the previously reported two step process of hydrolysis to form HI and oxidation of HI by O_2_ to form I_2_.^[^ ^4^, ^5^ ^]^

Evidence that I_2_ gas formed when those parts of the perovskite layer not protected by the electrode degrade does not then corrode the Bi capped Cu electrode is provided by the following experiment: The top four layers of the device stack (i.e., C_60_|BCP|Cu|Bi) were deposited onto a glass substrate with the C_60_|BCP layer deposited over the whole glass surface and the Cu|Bi electrodes deposited as three separate 2 mm wide strips. The Cu|Bi strip electrodes were 10 mm in length and electrical contacts were made at either end of each strip with contact to the Cu layer as schematically shown in the Figure S11 (Supporting Information). Adjacent to this was placed a second glass substrate of the same area, coated with a perovskite and C_60_ bilayer (Figure S11, Supporting Information). The resistivity of Cu (1.72 × 10^−8^ Ω m)^[^ ^32^ ^]^ is two orders of magnitude lower than that of Bi (1.29 × 10^−6^ Ω m)^[^ ^33^ ^]^ and the thickness of the Cu layer is three times greater than the Bi layer, so the conductance of the bilayer electrode is dominated by that of the Cu layer. Consequently, conversion of Cu to CuI due to reaction with I_2_ gas would manifest as an increase in the resistance of the bilayer electrode due to the very high resistivity of CuI (72 × 10^−5^ Ω m)^[^ ^34^ ^]^ as compared to Cu.^[^ ^6^ ^]^ Both substrates were light soaked under 1 sun simulated solar illumination in air for 20 h, over which time the perovskite layer completely decomposed to a transparent yellow color (Figure S11, Supporting Information). At the end of this period the resistance of the Cu|Bi strip electrodes was reduced by between 21% and 30%: Table S4 (Supporting Information). This counterintuitive result is attributed to a reduction in the resistance of the Cu film because we have previously shown that the resistance of 100 nm Cu film under constant 1 sun simulated illumination for 20 h in ambient air decreases by 12.9% ± 6.2%.^[^ ^6^ ^]^ However, in that previous study the Cu film was not protected from oxidation – a process that inevitably increases the sheet resistance^[^ ^35^, ^36^ ^]^ – which explains why the absolution reduction in resistance is smaller than observed for the Bi capped Cu film. It has also been reported that the resistance of Cu grids, fabricated by lithographically patterning evaporated Cu films, reduces by ≈25% upon storage in a nitrogen filled glovebox at ambient temperature for a period of weeks^[^ ^37^ ^]^ and that the same reduction in resistance can be achieved by low temperature heating (120 °C) for a short time (15 min),^[^ ^38^ ^]^ which indicates that the process that gives rise to the reduction in resistance is thermally activated. The X‐ray diffraction (XRD) pattern for Cu films without a Bi capping layer before and after light soaking; Figure S12 (Supporting Information), show no evidence of a substantive change in the crystal structure of the film upon light soaking. However, it is known that 100 nm Cu films deposited by thermal evaporation are polycrystalline with a small mean crystallite diameter of ≈20 nm,^[^ ^6^ ^]^ so it is likely that the primary determinant of the in plane electrical resistance is the contact resistance between adjacent crystallites, resulting from scattering of electrons as they traverse the interfaces. It has been reported that Cu atoms at the surface of Cu nanoparticles with diameters in the range 6–17 nm are mobile at room temperature,^[^ ^39^ ^]^ so it is tentatively suggested that in the current context the electrical contact between adjacent crystallites improves as a result of Cu atom rearrangement at the interface between crystallites to alleviate strain – a scale of atom rearrangement not easily detected by XRD. Regardless of the underlying mechanism, while it is possible that the large reduction in resistance due to this phenomenon could mask an increase in resistance resulting from Cu corrosion by I_2_, it is reasonable to conclude that substantive conversion of Cu to CuI can be ruled out.

The TEM, SEM, and EDX results show that the Bi layer not only passivates the Cu cathode from chemical attack by I_2_ gas, but also efficiently sequesters I_2_ in the form of a solid film on top of the Bi layer. To shed light on the underlying reason for the latter a Bi film supported on Cu together with gold film (100 nm) on glass and a silicon wafer were simultaneously exposed to I_2_ gas produced by heating iodine crystals in ambient air. EDX elemental analysis was then used to compare the relative uptake of I_2_. A gold film and silicon wafer were chosen because, like Bi, they do not react with I_2_ gas in air at ambient temperature: See Table S1 (Supporting Information) and reference 40. It is evident from the EDX spectra in Figure S13 (Supporting Information) that I_2_ deposition only occurs on the Bi substrate, which supports the conclusion that I_2_ has a particularly high affinity for Bi films. In the absence of a chemical reaction between a molecule in the vapor phase and a solid surface, vapor deposition is expected to occur when the potential well depth associated with the attractive van der Waals interactions between the substrate and molecule exceeds the kinetic energy of the molecule in the gas phase. Since there is no activation energy to physisorption of molecules at surfaces, equilibrium between molecules in the gas phase and those physisorbed on the substrate is expected to establish rapidly.^[^ ^40^ ^]^ In the absence of a chemical interaction with the substrate, the barrier to surface diffusion can be sufficiently small for physisorbed small molecules to diffuse at room temperature enabling crystalline assemblies of physisorbed molecules to form, which minimizes their collective energy.^[^ ^40^ ^]^ Crystal growth in the direction normal to the substrate can then ensue if the kinetic energy of molecules in the vapor phase is smaller than the potential well depth associated with physisorption onto the first layer. I_2_ is a highly polarisable molecule^[^ ^41^ ^]^ that forms a molecular solid with halogen bonding interactions between molecules being the dominant force of cohesion.^[^ ^42^ ^]^ The large electron density and *s^2^* lone pair on Bi also make it highly polarisable, even in the 3+ oxidation state.^[^ ^43^ ^]^ Indeed the polarizability of elemental Bi is ∼30% larger than Au^[^ ^43^ ^]^ and the polarizability of Bi^3+^ in Bi_2_O_3_ is ≈2.5 times greater than that of Si^4+^ in SiO_2_.^[^ ^43^ ^]^ Since dispersive force interactions scale with the polarizability of the interacting species, the interaction strength between Bi and I_2_ molecules in the vapor phase (either in its metallic or oxide form) is expected to be much greater than that between I_2_ and metallic Au or SiO_2_, which offers a plausible explanation for the much higher affinity of I_2_ for Bi than Au or the SiO_2_ surface of silicon.

**Figure 3:**
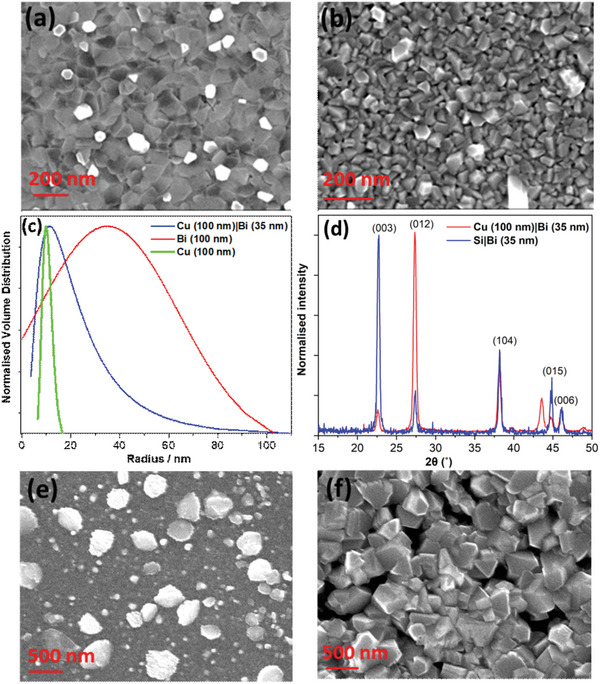
a) SEM image of Bi film supported on a Si wafer; b) SEM image of Bi film supported on Cu; c) Crystallite size distribution extracted from SAXS analysis; d) Grazing incidence XRD patterns of Bi films supported on Si wafer and Cu; e) SEM image of Iodine adsorbed on to Bi film supported on a Si wafer; f) SEM image of Iodine adsorbed on to Bi film supported on Cu. The peak at 43.5° in the XRD pattern is the peak corresponding to Cu (111) plane.

In order to elucidate the importance, if any, of the Cu film underneath the Bi film in enabling I_2_ gas adsorption, it was necessary to determine the structure of Bi film on Cu as compared to a Bi film deposited onto a substrate with which it is expected to have no chemical interaction. For the latter, silicon wafer was chosen since it is terminated with a native oxide layer that is well known to interact weakly with condensing metals.^[^ ^35^, ^44^ ^]^ The SEM images in **Figure** **3**a,b together with the atomic force microscopy (AFM) images in Figure S14 (Supporting Information) and the results of a grazing incidence small‐angle X‐ray scattering (GISAXS) study given in Figure 3c, show that the average crystallite size for a 35 nm thick Bi film deposited onto Cu is much smaller than that on a silicon wafer; mean ≈19 nm versus ≈35 nm, with a much narrower size distribution. The smaller crystallite size on Cu is consistent with a stronger interaction between the condensing Bi atoms and the receiving substrate than between Bi and SiO_2_, because diffusion of a more tightly bound adsorbate will be suppressed giving rise to a higher density of crystallite nucleation sites.^[^ ^45^ ^]^ It is evident from Figure 3c that the mean crystallite size for Bi on Cu is similar to that of the underlying Cu, which is further evidence for a strong interaction between the condensing Bi atoms and the receiving Cu substrate because the Cu surface is templating the growth of the Bi crystallites. It is concluded that the metallic bonding interaction between condensing Bi atoms and the Cu substrate is the reason for the suppressed diffusion. The grazing incidence XRD patterns in Figure 3d and powder diffraction patterns of the same films; Figure S15 (Supporting Information), show that 35 nm Bi films on silicon and Cu are polycrystalline, since there are peaks corresponding to reflection from the (003), (012), (104), (015), and (006) crystal planes. In the powder diffraction pattern of Bi on Cu (Figure S15, Supporting Information) the most intense peak corresponds to reflection from the (012) plane, which indicates that this is the preferred crystallite orientation. In contrast, for Bi deposited onto a silicon wafer the preferred orientation of Bi crystallites is (003) because this reflection is the most intense. This large difference in preferred orientation is consistent with a significant difference in the strength of interaction between Bi and the underlying substrate.

Given the significant difference in crystallite size and index for Bi films supported on Cu and on silicon wafer (with native oxide), a comparative study of the ability of these two types of Bi film to take up I_2_ gas was performed by exposing both films to I_2_ gas formed by sublimation from iodine crystallites, and comparing the intensity of the iodine peaks in the EDX spectra. Remarkably, while both films readily adsorb I_2_ gas, the intensity of the iodine peak in the EDX spectrum of Bi/Cu is an order of magnitude greater than that on silicon wafer; Figure S16 (Supporting Information). SEM imaging; Figure 3f, shows that iodine forms a dense polycrystalline layer on Cu/Bi film with crystallite dimensions much larger than that of the underlying metal. In contrast, I_2_ adsorbed on the Bi film supported on a silicon wafer; Figure 3e, appears discontinuous with localized platelets of condensed iodine. While surface roughness can play a role in seeding crystallite nucleation, in this case, the Cu/Bi and Bi films have a comparable root‐mean‐square roughness of 3.7 ± 0.1 nm and 3.9 ± 0.2 nm, respectively (measured over an area of 2 × 2 microns). There is however a strong correlation between the coverage of the Bi film by iodine crystals and the prevalence of the (012) crystal face as measured using XRD; Figure S15 (Supporting Information). The peak corresponding to reflection from the (012) crystal face is the dominant peak in the XRD pattern for Bi on Cu. The peak corresponding to reflection from the lowest energy face, (003),^[^ ^46^ ^]^ is a small fraction of the intensity of the (012) reflection. The relative intensity of these peaks is strikingly reversed in the diffraction pattern of Bi on silicon wafer: Figure S15 (Supporting Information). Given that the surface of the oxide formed on these different crystal faces of Bi will almost certainly have different densities and arrangements of Bi and oxygen atoms, the magnitude of the attractive interaction between the first layer of I_2_ molecules and these two oxide surfaces will likely be different. Similarly, the ease with which physisorbed I_2_ molecules can assemble into the crystalline domains needed to seed crystal growth normal to the oxide surface is also likely to be different. It is therefore plausible that the balance of interactions at the surface of the oxide layer formed on Bi (012) is more favorable for I_2_ adsorption and crystallization. Computational studies are underway to validate this hypothesis.

The device stability studies, nanoscale elemental analysis of a cross‐section of the Cu / Bi electrode and electrical measurements of Cu / Bi electrode show that the 35 nm Bi capping layer is capable of protecting the Cu cathode from oxidation by I_2_ gas released by the degradation of the that part of the perovskite not protected by the cathode upon exposure to ambient air. To reconcile these findings with the initial observation that for Bi thickness ≥ 35 nm degradation of the underlying Cu still occurs when exposed to a continuous flux of I_2_ gas produced by heating iodine crystals in air; Figure S1 (Supporting Information), it is necessary to consider the role of pinholes in evaporated metal films film. Pinholes in evaporated films result from the presence of dust particles on the receiving substrate, which are unavoidable in our laboratory because glass processing was not undertaken in a clean room environment. The directional nature of vacuum evaporation means there is inevitably a shadowing effect around dust particles, so complete coverage of the Cu cathode cannot be guaranteed even when the edges of the film are sealed with epoxy resin. When metal films are subjected to a continuous flow of I_2_ vapor over the whole film surface – as was the case in the initial corrosion test conducted part of this study – sufficient I_2_ gas ultimately diffuses through pinholes to corrode the underlying Cu film, as is observed to be the case from the edges of the 100 nm thick Cu capped with Bi. However, in the context of PPV devices studies I_2_ gas is only produced by decomposition of that part of the perovskite layer not capped by the cathode, which is a much more limited dose of I_2_, and we have shown that it is primarily sequestered near to the edges of the cathode, so the adverse effect of random pinholes in the Bi layer is far less significant.

## 3 Conclusion

In summary, we have shown that a Bi capping layer on top of the copper cathode in inverted organo‐tin halide PPVs is an exceptionally effective and simple to implement means of improving the operational stability of this emerging class of PPV device toward degradation in air. Corrosion of the Cu cathode by iodine gas (produced when those parts of the perovskite layer that are not protected by the electrode degrade) is blocked by the Bi layer and is sequestered as a thin solid film on top of the device. Bi is well‐matched to this application because it is an environmentally benign, non‐toxic, and low‐cost material. In principle, this approach can also be applied in PPV devices using a Ag top electrode, although Cu has the advantage that it is ≈1% of the cost of Ag with comparable electrical conductivity. The electrode‐based approach to improving device stability reported herein sets the stage for major further improvements in the stability of organo‐tin halide PPVs, since it can be combined with any of the recently reported chemical approaches to improving the intrinsic stability of organo‐tin halide perovskites in air.

## 4 Experimental Section

### Substrate Cleaning

Substrates were cleaned by ultrasonic agitation in a dilute solution of surfactant followed by deionized water, acetone, and isopropyl alchol (IPA) for 15 min each. They were then dried using a stream of nitrogen and were UV‐O_3_ treated for 15 min immediately before use.

### Device Fabrication

PEDOT:PSS (Al 4083) was deposited onto freshly UV‐O_3_ treated ITO by dropping onto the ITO so as to cover the entire substrate, then spinning at 5000 rpm for 60 s in ambient air followed by a 10 min annealing at 120 ˚C. Then these electrodes were transferred to a nitrogen‐filled glovebox (≤ 1 ppm O_2_ and H_2_O) for the rest of device fabrication. FA_0.78_GA_0.2_SnI_3_‐1% EDAI_2_ + 10 mol % SnF_2_ from DMSO was spin coated at 5000 rpm for 90 s. Chlorobenzene (600 µL) was dropped onto the still wet perovskite film 35 s after the start of spin coating. The resulting perovskite film was annealed at 70 ˚C for 15 min to drive off residual DMSO. The film was then transferred to a vacuum evaporator co‐located in the same glovebox and a 32.5 nm layer of C_60_ was deposited by thermal evaporation at a rate of 0.1–0.4 Å s^−1^. The device was completed by evaporating a 5 nm BCP layer at a rate of 0.6–0.7 Å s^−1^ followed by 100 nm of Cu at 1–1.5 Å s^−1^ and 35 nm of Bi at 1–1.5 Å s^−1^. It is important to note that Cu and Bi should be evaporated from the same source to ensure that there are no shadowing effects. Thermal evaporations were performed at a pressure of 2 × 10^−6^ mbar with substrate rotation. The Cu|Bi electrode was deposited through a shadow mask to make six devices per slide, each with an area of 6 mm^2^.

### Device Testing

Device testing was performed in the same nitrogen filled glove box as used for device fabrication (≤ 1 ppm O_2_ and H_2_O) using an ABET Technologies Sun 2000 solar simulator. Current density–voltage (*J‐*‐*V*) curves were measured using a Keithley 2400 source‐meter under AM1.5 G solar illumination at 100 mW cm^−2^ (1 sun), scanned from −0.2 to + 1 V at 0.1 Vs^−1^. *J–V* measurements were made using custom LabVIEW program. Stability tests were performed under continuous 1 sun simulated solar illumination (Xenon short arc lamp, AM1.5 G solar illumination at 100 mW cm^−2^) with the device under load at a fixed voltage bias close to the maximum‐power‐point. During device testing the temperature of the device increases to ≈43^o^ as measured using a thermocouple mounted in a calibrated silicon solar cell located at the same height and immediately adjacent to the PPV device being tested.

### Electrode Stability Studies

Substrates were prepared by evaporating 2 mm × 12 mm strips of 100 nm of Bi|Cu (or Cu) supported on to C_60_ (32.5 nm) and BCP (5 nm) on a glass substrate. All the electrodes were subjected to continuous 1 sun simulated solar illumination in ambient air and the resistance was measured at regular intervals. The number of samples of each type was four and the resistance was measured using the 2‐point probe method. Electrical contact was made using two parallel strips of Ag along the full length of rectangular samples. The distance between the parallel silver electrodes was measured using a caliper.

### Scanning Transmission Electron Microscopy (STEM)

Cross‐sectional TEM specimens were prepared using a focused ion beam. The specimens were observed and analyzed in a double aberration corrected JEOL ARM 200F TEM, equipped with a 100 mm^2^ Oxford Instruments windowless EDX detector.

### Atomic Force Microscopy (AFM)

AFM imaging was performed in tapping mode using an Asylum Research MFP‐3D to determine the step height of the films and morphologies.

### Scanning Electron Microscopy (SEM)

SEM imaging was performed using a Zeiss Gemini 55VP field emission gun SEM. EDXS spectra were recorded using an Oxford Instruments Si‐Li detector unit on the SEM instrument, at an accelerating voltage of 20 keV.

### GISAXS

Grazing incidence small‐angle X‐ray scattering (GISAXS) measurements were made using a Xenocs Xeuss 2.0 equipped with a micro‐focus Cu K*α* source collimated with Scatter less slits and Pilatus 300k detector. (Full details are given in Supporting Information accompanying this paper). The Si|Cu (100 nm)|Bi (35 nm) film was modeled with a log normal distribution of spheres and Si|Bi (35 nm) films were modeled and fitted to a Gaussian distribution. Si|Cu (100 nm)|Bi (35 nm) film was modeled as a log normal distribution of spheres and the mean crystallite size was 19.33 ± 0.07 nm with a full‐width half maximum of 22.08 nm. Si|Bi (35 nm) film was modeled and fitted to a Gaussian distribution and the Gaussian mean crystallite size was 34.95 nm with a full‐width half maximum of 69.02 nm.

### XRD

XRD measurements were made on a 3rd generation Malvern Panalytical Empyrean equipped with multicore (iCore/dCore) optics and a Pixcel3D detector operating in 1D receiving slit mode. A Cu tube was used giving Cu Ka_1/2_ radiation (1.5419 Å). Grazing incidence measurements with an incidence angle of 0.5° were made in the range 15 – 90° 2*θ* with a step size of 0.04° and a counting time of ≈1.4 s per step.

## Conflict of Interest

The authors declare no conflict of interest.

## Author Contributions

The manuscript was written through contributions of all authors. All authors have given approval to the final version of the manuscript.

## Supporting information

Supporting Information

## Data Availability

The data that support the findings of this study are available in the supplementary material of this article.
