# Supplementary material for: Highly Air Stable Tin Halide Perovskite Photovoltaics using a Bismuth Capped Copper Top Electrode
Source: Adv Sci (Weinh). 2023 Jun 16;10(24):2301497. doi: 10.1002/advs.202301497 (PMC10460886; doi:10.1002/advs.202301497)
Supplement: Supplementary file 1 — Supporting Information [file ADVS-10-2301497-s001.pdf]

ADVANCED SCIENCE
Open Access

Supporting Information

for *Adv. Sci.*, DOI 10.1002/advs.202301497

Highly Air Stable Tin Halide Perovskite Photovoltaics using a Bismuth Capped Copper Top Electrode

*Anjana Wijesekara, Yisong Han, David Walker, Steven Huband and Ross Hatton**

Highly Air Stable Organo-tin Perovskite Photovoltaics using a Bismuth Capped Copper Top Electrode

Anjana Wijesekara[1], *Yisong Han*[2], *David Walker*[2], *Steven Huband*[2] *and Ross Hatton*[1]*

[1]Department of Chemistry, University of Warwick, CV4 7AL, Coventry, United Kingdom.

[2]Department of Physics, University of Warwick, CV4 7AL, Coventry, United Kingdom.

*Corresponding Author. E-mail: Ross.Hatton@warwick.ac.uk

Supporting information

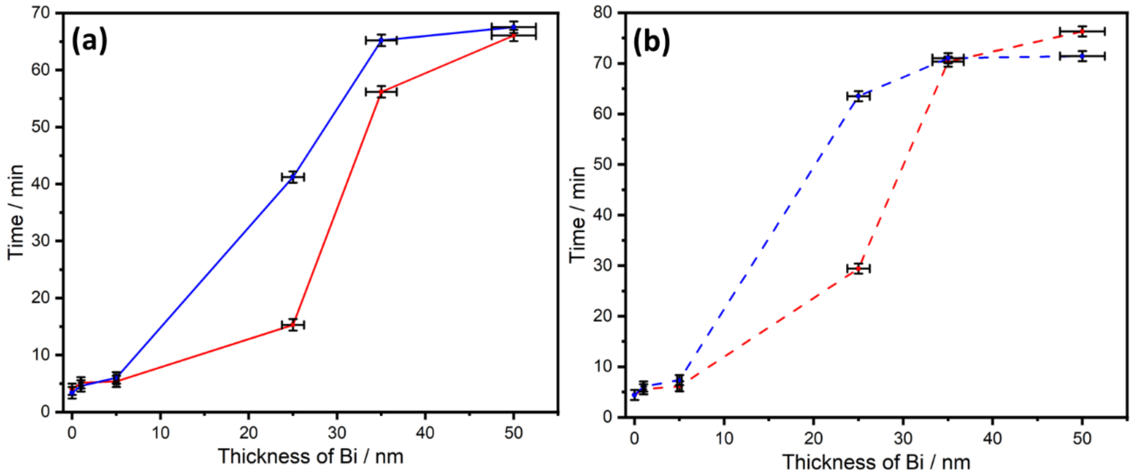

Figure S1| (a) Time taken for the onset of visible corrosion of a 100 nm Cu film supported on glass, as a function of the thickness of the bismuth (Bi) capping layer, when exposed to iodine vapour. **(b)** Time taken for the complete conversion of a 100 nm thick Cu film supported on glass into CuI, as a function of the thickness of the bismuth (Bi) capping layer, when exposed to iodine vapour.

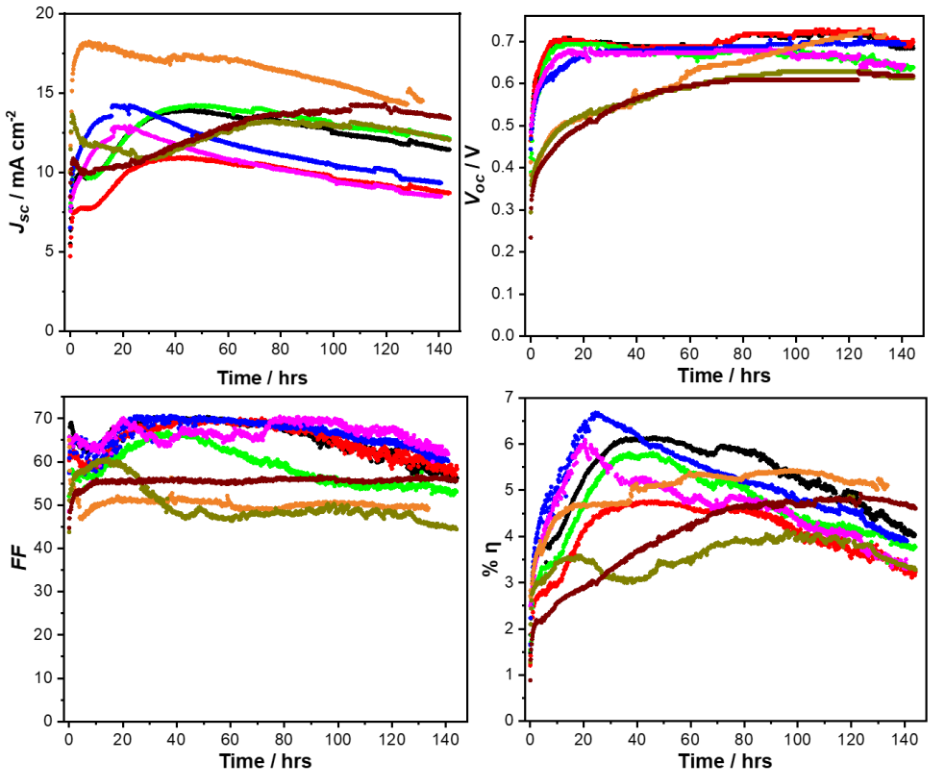

Figure S2| Evolution of the J_{SC}, V_{OC}, FF and η for unencapsulated perovskite PV devices with the architecture ITO|PEDOT:PSS (Al 4083)|perovskite|C_{60}|BCP|Cu|Bi tested in ambient air (relative humidity 30-70%) under continuous 1 sun simulated solar illumination with the device under load at (or very close to) maximum-power-point. Perovskite: $FA_{0.78}GA_{0.2}SnI_3$-1% $EDAI_2$ with 10 mol % SnF_2.

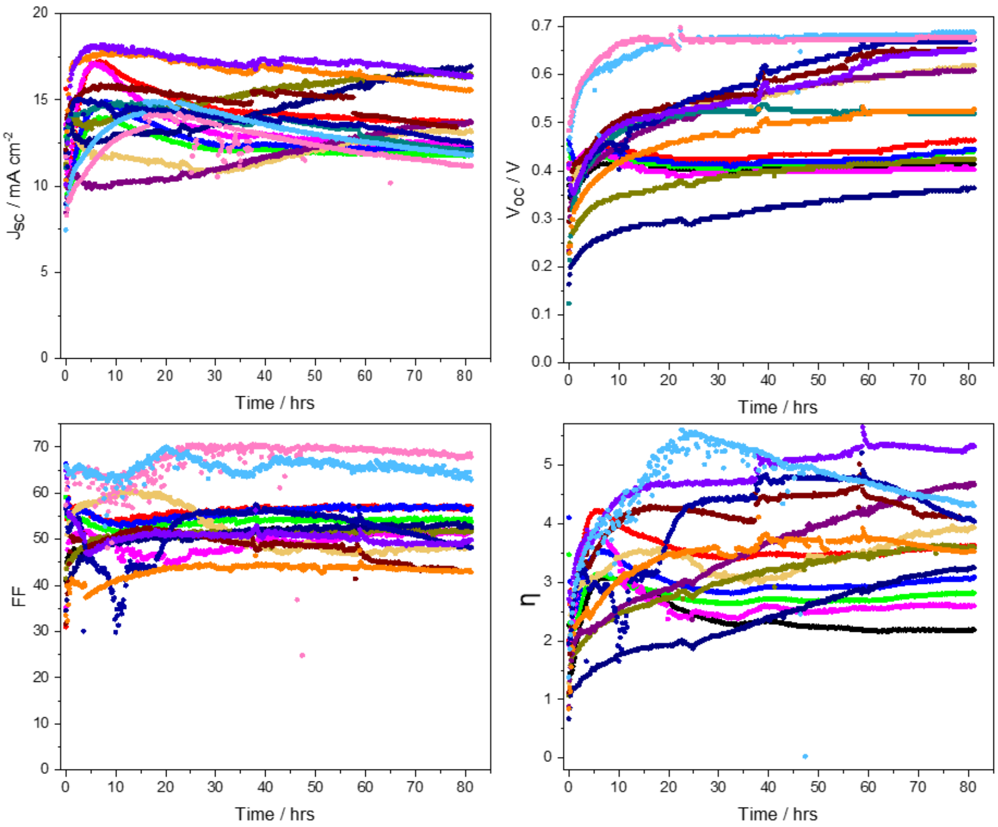

Figure S3| Evolution of the J_{SC}, V_{OC}, FF and η for unencapsulated perovskite PV devices with the architecture ITO|PEDOT:PSS (Al 4083)|perovskite|C_{60}|BCP|Cu|Bi tested in ambient air (relative humidity 30-70%) under continuous 1 sun simulated solar illumination with the device under load at (or very close to) maximum-power-point. Perovskite: $FA_{0.78}GA_{0.2}SnI_3$-1% $EDAI_2$ with 10 mol % SnF_2.

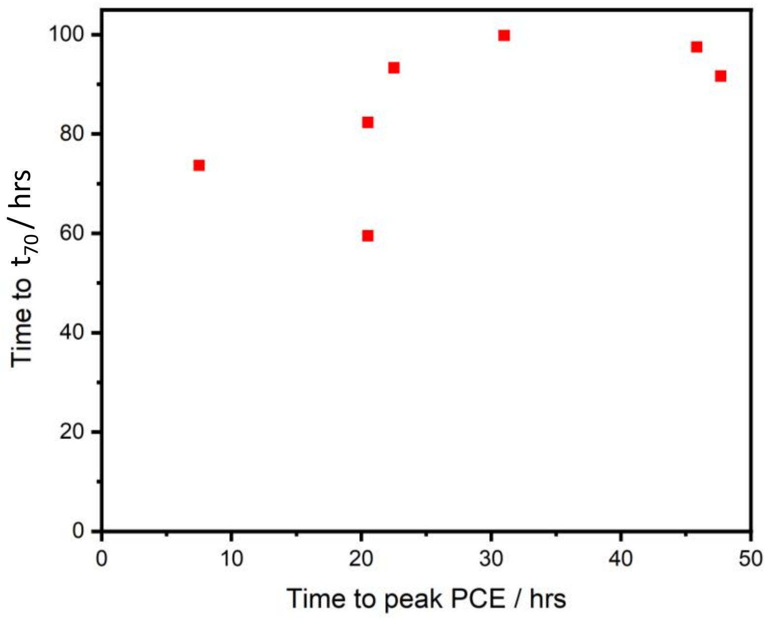

Figure S4| Plot of the time taken to achieve peak power conversion efficiency (PCE) *vs* time taken for the PCE to reduce to 70% of its peak value (t_{70}), for unencapsulated perovskite PV devices with the architecture ITO|PEDOT:PSS (Al 4083)|perovskite|C_{60}|BCP|Cu|Bi tested in ambient air (relative humidity 30-70%) under continuous 1 sun simulated solar illumination with the device under load at (or very close to) maximum-power-point. Perovskite: $FA_{0.78}GA_{0.2}SnI_3$-1% $EDAI_2$ with 10 mol % SnF_2.

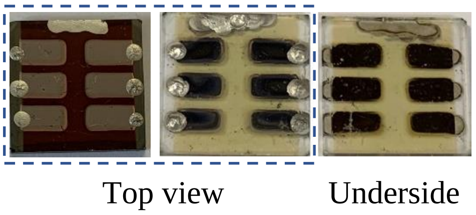

Figure S5| Representative photographs of PPV devices supported on 1.44 cm^2 glass substrates before (left) and after (middle and right) degradation in air. The colour change from deep red to transparent yellow in those areas not covered by the cathode is indicative of perovskite degradation.

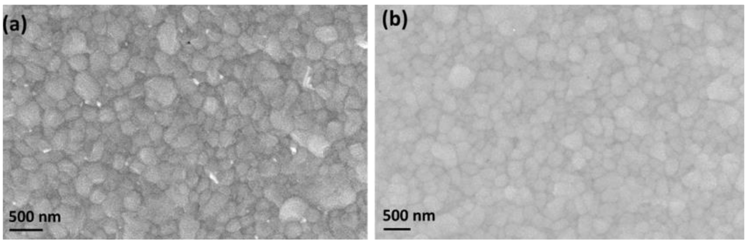

Figure S6| SEM images of the perovskite film underneath the electrode after removal of the electrode and the organic semiconductor layers: **(a)** aged device (145 hours testing from peak power conversion efficiency) and **(b)** fresh device (i.e. not tested).

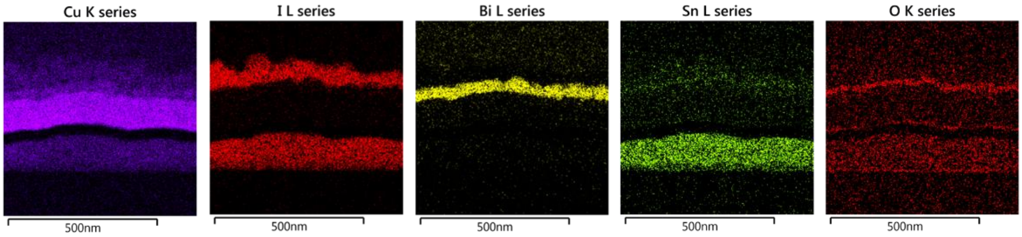

Figure S7| EDX analysis elemental maps for the cross-sectional TEM image shown in Figure 2 of the main manuscript. The cross-section was taken from near to the edge of the Cu / Bi cathode of an aged PPV device with the structure: ITO|PEDOT:PSS (Al 4083)|perovskite|C_{60}|BCP|Cu|Bi.

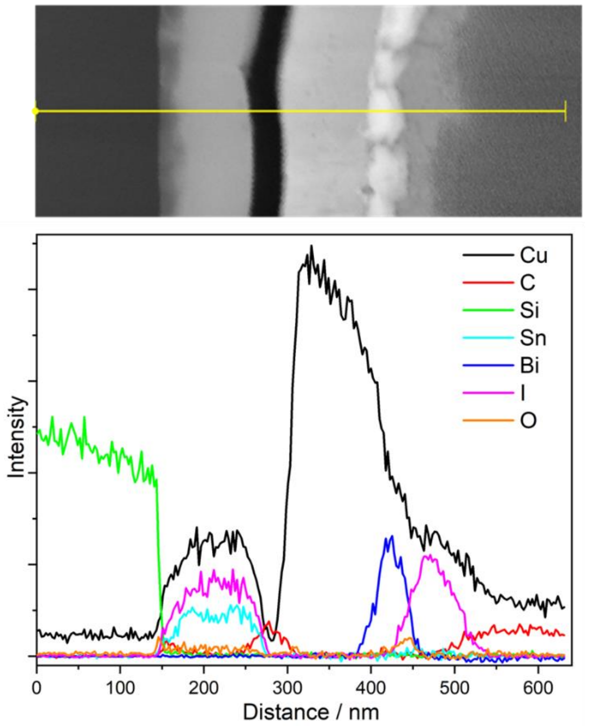

Figure S8| TEM cross-section and EDX line profile along the yellow line indicated in the TEM image. Please note that the sample cross-section is mounted on a Cu grid for TEM analysis and so the Cu signal is not a true representation of the Cu distribution across the sample.

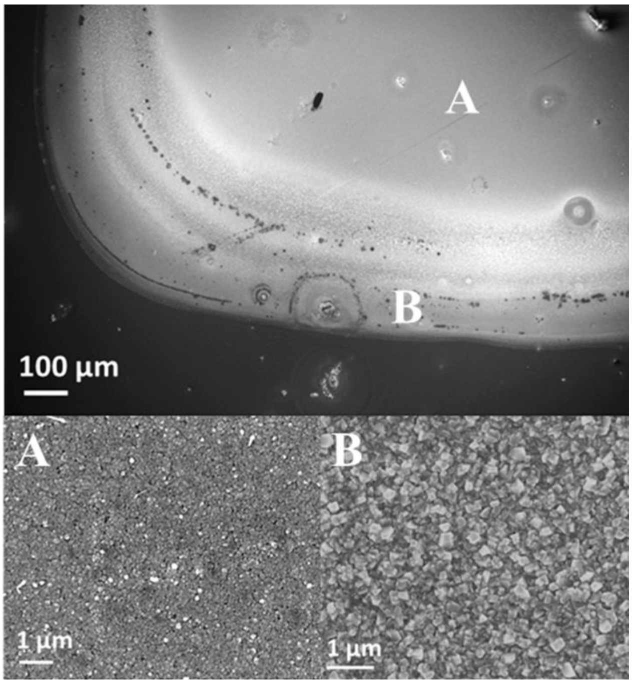

Figure S9| Upper: Large area SEM image showing part of a PPV device cathode after stability testing with middle and edge areas indicated by A and B respectively. **Lower:** Representative higher resolution SEM images taken from the middle (A) and the edge (B).

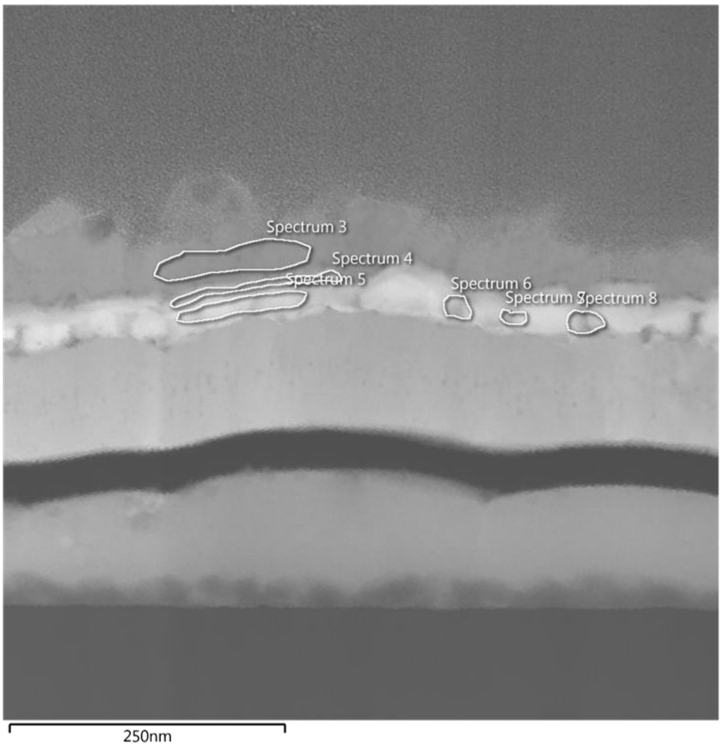

Element	Spectrum 3	Spectrum 4	Spectrum 5	Spectrum 6	Spectrum 7	Spectrum 8
C	5.94	11.36	3.05	16.49	10.19	5.26
O	1.30	27.92	6.44	8.33	6.69	9.50
Al	2.91	2.88	4.22	0.00	0.00	0.00
Si	19.93	0.00	0.00	10.61	0.00	24.20
Cu	42.60	39.24	58.69	44.89	48.84	40.73
Sn	0.89	0.70	0.00	0.00	0.00	0.36
I	26.38	2.40	0.85	1.45	0.00	0.68
Bi	0.06	15.51	26.76	18.23	34.28	19.27
Total	100.00	100.00	100.00	100.00	100.00	100.00

Figure S10| Elemental compositions (atomic percentages) determined using EDX at the locations indicated in TEM image above. The composition of the oxide layer is estimated from spectrum 4.

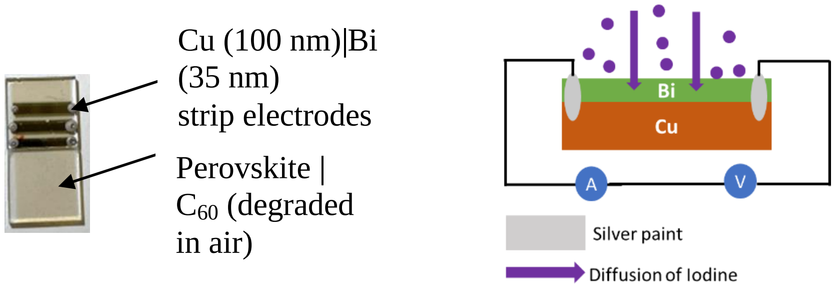

Figure S11| (Left) Photograph and the schematic (**Right**) of the experimental set-up used for monitoring the resistance change of bismuth capped copper electrodes exposed to I_2 gas emanating from an adjacent decomposing perovskite film.

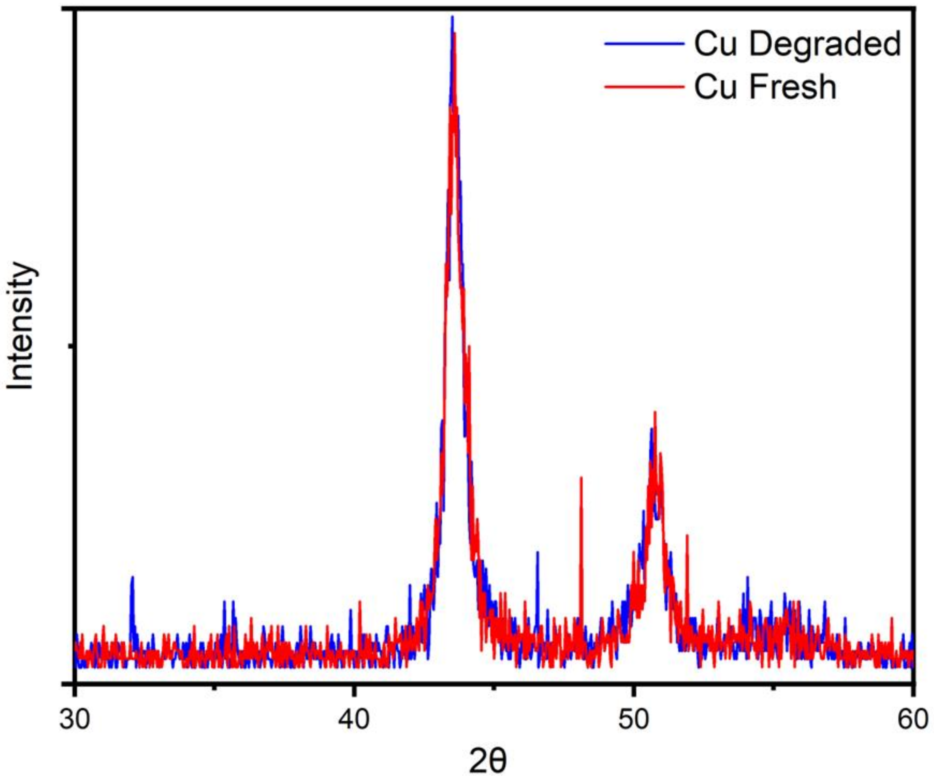

Figure S12| XRD pattern of a 100 nm Cu film supported on a Si wafer coated with a C_{60} (32.5nm) layer and BCP (5 nm) layer, before and after exposing to constant illumination for 20 hours in ambient air.

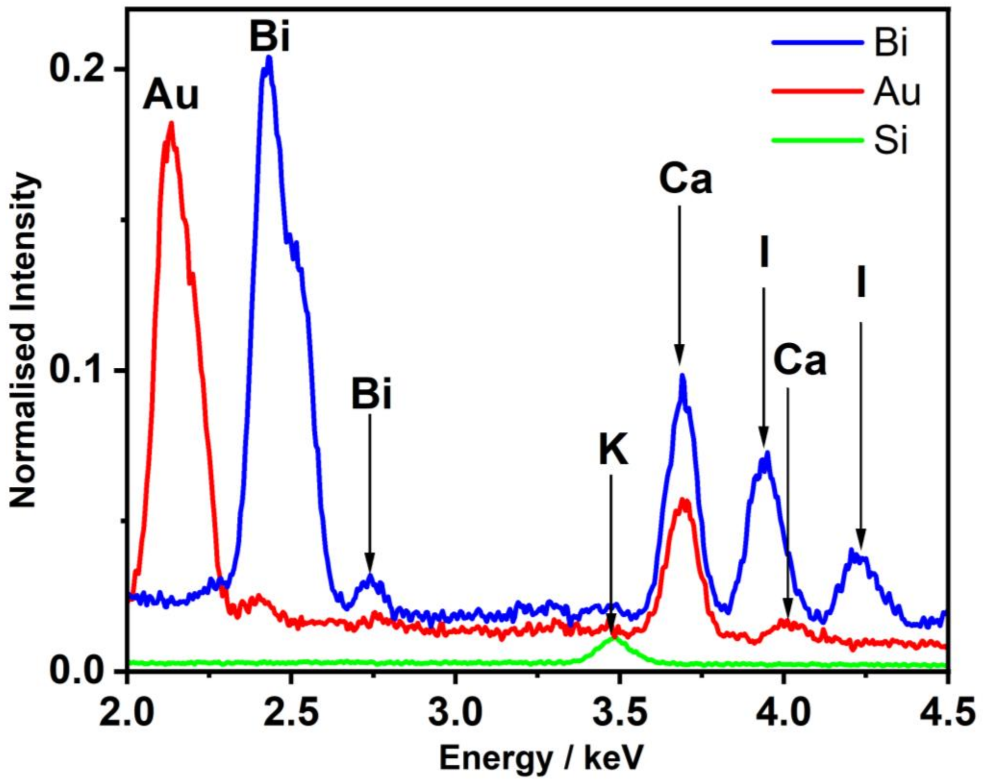

Figure S13| EDX spectra of a 35 nm Bi film on Cu (Blue), 100 nm Au on glass (Red) and silicon wafer with native oxide (Green) after exposure to the same I_2 gas environment for the same duration.

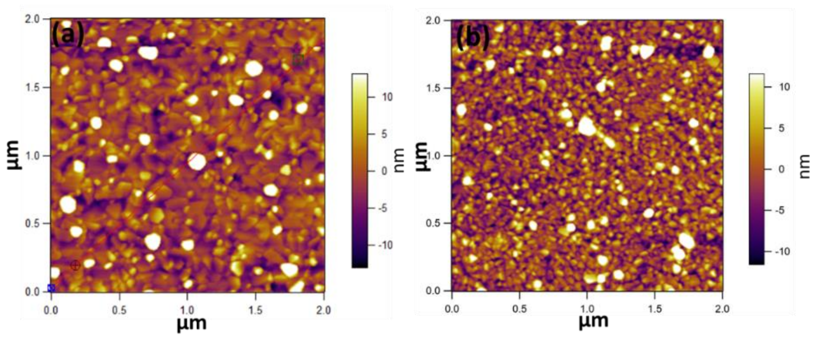

Figure S14| AFM image of **(a)** Bi film (35 nm) supported on Si wafer; **(b)** Bi film (35 nm) supported on Cu (100 nm) on glass.

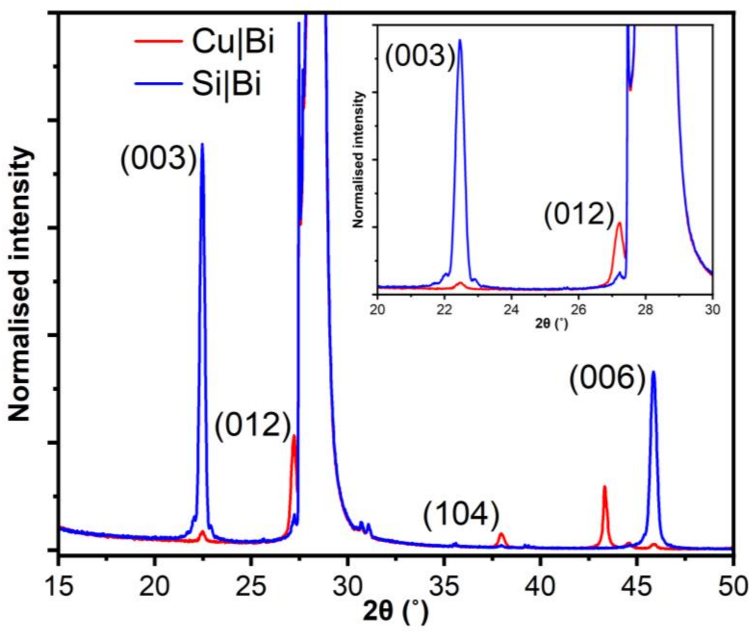

Figure S15| Powder X-ray diffraction patterns of Bi films (35 nm) supported on silicon wafer (blue) and Cu (100 nm) coated Si wafer (red). The very intense peak at ~ 28.5^{o} and the peak at ~ 43° result from the underlying single-crystal silicon substrate and polycrystalline Cu film respectively.

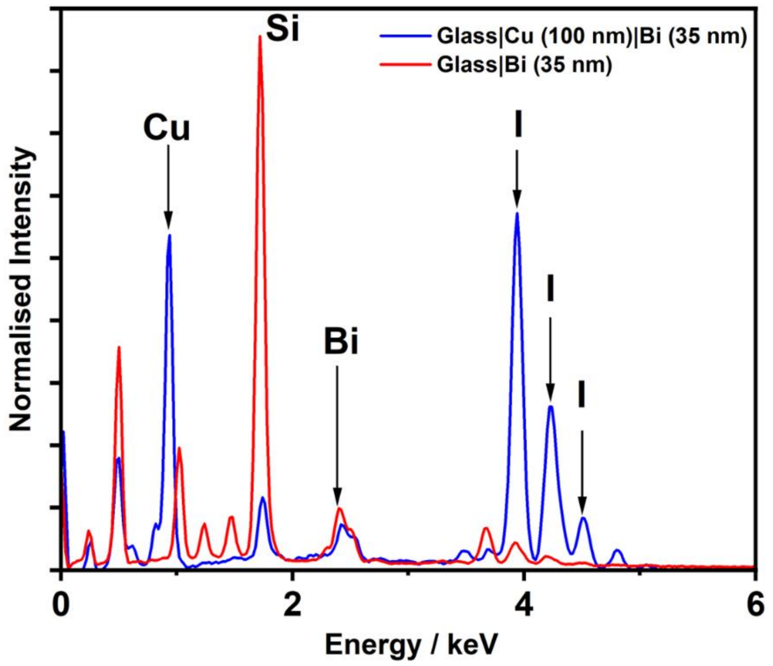

Figure S16| EDX spectra of a 35 nm Bi film on Cu (Blue) and 35 nm Bi film on a silicon wafer after exposure to the same I_2 gas environment for the same duration.

Table S1| Summary of the bond energies and standard enthalpies for common elements.[1-4]

Material	Metal - Metal (E_{M-M}) (kJ/mol)	Metal - Iodine (E_{M-I}) (kJ/mol)	Energy barrier ($\Delta E = E_{M-M} - E_{M-I}$) (kJ/mol)	Standard enthalpy of formation ($\Delta_f H$, 298.15K) (kJ/mol)
**Ag**	162.9	234	-71.1	-61.8 (AgI)
**Al**	264.3	369.9	-105.6	-302.9 (AlI_3)
**Au**	226.2	276	-49.8	0 (AuI)
**Bi**	204.4	186.1	18.3	-159.69 (BiI_3)
**Mo**	435.5	266.9	168.6	-103.9 (MoI_2)
**Cd**	7.36	97.2	-89.84	-203.3 (CdI_2)
**Co**	127	280	-153	-88.7 (CoI_2)
**Cr**	152	287	-135	-205 (CrI_3)
**Cu**	201	289	-88	-68.7 (CuI)
**Fe**	118	123	-5	-113 (FeI_2)
**Sn**	187	235	-48	-143.5 (SnI_2)
**In**	82	306.9	-224.9	-238 (InI_3)
**Ni**	204	293	-89	-78.2 (NiI_2)
**Ti**	117.6	306	-188.4	-375.7 (TiI_4)
**Mg**	11.3	229	-217.7	-364 (MgI_2)
**Zn**	22.2	153.1	-130.9	-208 (ZnI_2)
**Pb**	86.6	194	-107.4	-175.5 (PbI_2)

Table S2| Stability of unencapsulated organo-tin perovskite PV devices tested in ambient air under 1 sun continuous simulated solar illumination reported in the literature to date.

Perovskite	Conditions<br>RH = Relative Humidity	Time / hrs	Power conversion efficiency retained after test / %	Reference
$FASnI_3$	Light soaking<br>RH 20%	16	50%	[5]
$FASnI_3$	RH 20%<br>Light soaking	10	60%	[6]
$FASnI_3$	RH 20%<br>Light soaking	2.3	70%	[7]
$FA_{0.78}GA_{0.2}SnI_3$	RH 30-50%<br>Under load	44.5	70%	[8]
$FASnI_3$<br>$+NaBH_4$+ DiPI	RH 60%<br>Under load (0.45 V)	9	80%	[9]
$FA_{0.78}GA_{0.2}SnI_3$	RH 30%-70%<br>Under load	100<br>(78)	70%<br>(80%)	This work

Table S3| The Sn:I ratio at the edge and centre of the cathode of a degraded PPV device determined using spatially resolved EDX analysis. The edge and middle regions are indicated in Figure S8.

	Sn	I
**Edge**	1	6.26
	1	8.58
	1	10.54
	1	8.42
	1	8.06
	1	11.30
	1	17.54
**Centre**	1	4.38
	1	4.78
	1	3.40
	1	4.23
	1	3.52

Table S4| Two point electrical resistance of strips (2 mm wide and 10 mm long) of Cu (100 nm) | Bi (35 nm) supported on glass substrates pre-coated with C_{60}|BCP layer. Electrical contact was made at either end of each strip with contact to the Bi and Cu layers. Immediately adjacent was placed a second glass substrate of the same area, coated with a perovskite and C_{60} bilayer. All substrates were then light soaked under 1 sun simulated solar illumination in air for 20 hours, over which time the perovskite layer completely decomposed to a transparent yellow colour forming a cloud of I_2 gas that diffuses over the slides with Cu|Bi strips.

**Sample**	***t*** **= 0 hrs Resistance / Ω ± 0.05Ω**	***t*** **= 6 hrs Resistance / Ω ± 0.05Ω**	***t*** **= 20 hrs Resistance / Ω ± 0.05Ω**
1			
Pixel 1	2.1	1.7	1.6
Pixel 2	2.0	1.7	1.7
Pixel 3	2.2	1.7	1.6
2			
Pixel 1	2.2	1.7	1.5
Pixel 2	2.2	1.7	1.5
Pixel 3	2.2	1.8	1.7
3			
Pixel 1	2.3	1.8	1.6
Pixel 2	2.1	2.3	2.3
Pixel 3	2.6	2.4	1.9
4			
Pixel 1	2.5	1.7	1.7
Pixel 2	2.1	2.0	1.8
Pixel 3	2.0	1.7	1.6

Experimental Procedures

Grazing incidence small-angle X-ray scattering (GISAXS) measurements were made using a Xenocs Xeuss 2.0 equipped with a micro-focus Cu Kα source collimated with Scatterless slits. The scattering was measured using a Pilatus 300k detector with a pixel size of 0.172

mm × 0.172 mm. The distance between the detector and the sample was calibrated using silver behenate ($AgC_{22}H_{43}O_2$), giving a value of 2.480(5) m. The magnitude of the scattering vector (q) is given by $q=4\pi \sin(\theta)/\lambda$, where 2θ is the angle between the incident and scattered X-rays and λ is the wavelength of the incident X-rays. This gave a q range for the detector of 0.003 Å-1 and 0.13 Å-1 in the horizontal plane. This q range allows crystallite sizes between 1 and 200 nm to be determined. Samples were aligned such that the surface was parallel to the beam and in the centre of the beam. To maximize the scattering signal from the Ag or Cu layers the sample was positioned at an incidence angle (αi) of 0.3° which is close to the critical angle of 0.4° for Ag or Cu with Cu Kα radiation. Integrating the in-plane scattering as a function of q allows the horizontal radius of the crystallites to be determined. The horizontal scattering was fitted using spheres with a lognormal distribution of the radius using the Irena analysis package.[10]

References

[1] S. Wu, R. Chen, S. Zhang, B. H. Babu, Y. Yue, H. Zhu, Z. Yang, C. Chen, W. Chen, Y. Huang, S. Fang, T. Liu, L. Han, W. Chen, *Nat. Commun*. **2019**, 10, 1.

[2] W. M. Haynes, CRC handbook of chemistry and physics, **2014**, 7.

[3] D. Cubicciotti, *Inorg. Chem.* **1968**, *7*, 7.

[4] M. W. Chase, J. L. Curnutt, H. Prophet, A. N. Syverud, L. C. Walker, *J. Phys. Chem. Ref. Data.* **2009**, 3, 311.

[5] Tai, Q., Guo, X., Tang, G., You, P., Ng, T. W., Shen, D., Cao, J., Liu, C. K., Wang, N., Zhu, Y., Lee, C. S., Yan, F. *Angew. Chemie - Int. Ed.* **58**, 806-810 (2019).

[6] Cao, J. Tai, Q., You, P., Tang, G., Wang, T., Wang, N., Yan, F. *J. Mater. Chem. A* **7**, 26580-26585 (2019).

[7] Wang, T., Tai, Q., Guo, X., Cao, J., Liu, C. K., Wang, N., Shen, D., Zhu, Y., Lee, C. S. Yan, F. *ACS Energy Lett.* **5**, 1741-1749 (2020).

[8] Wijesekara, A., Walker, M., Han, Y., Walker, D., Huband, S. & Hatton, R. A, *Adv. Energy Mater.* **11**, 1–9 (2021).

[9] Sanchez-Diaz, J., Sánchez, R. S., Masi, S., Krečmarová, M., Alvarez, A. O., Barea, E. M., Rodriguez-Romero, J., Chirvony, V. S., Sánchez-Royo, J. F., Martinez-Pastor, J. P. & Mora-Seró, I. *Joule* **6**, 861–883 (2022).

[10] J. Ilavsky, P. R. Jemian, J. Appl. Crystallogr. 2009, 42, 347.
